# 3-Sulfopropyl acrylate potassium-based polyelectrolyte hydrogels: sterilizable synthetic material for biomedical application

**DOI:** 10.1039/d4ra03901g

**Published:** 2024-09-11

**Authors:** Johanna Romischke, Thomas Eickner, Niels Grabow, Udo Kragl, Stefan Oschatz

**Affiliations:** a University of Rostock, Institute of Chemistry, Department of Industrial and Analytical Chemistry Albert-Einstein-Str. 3A Rostock 18059 Germany; b Rostock University Medical Center, Institute for Biomedical Engineering Friedrich-Barnewitz-Str. 4 18119 Rostock Germany stefan.oschatz@uni-rostock.de; c Department Life, Light & Matter (LLM), University of Rostock Rostock Germany

## Abstract

Hydrogels are extensively used in the biomedical field due to their highly valued properties, biocompatibility and antimicrobial activity and resistance to rheological stress. However, determining an efficient sterilization protocol that does not compromise the functional properties of hydrogels is one of the challenges researchers face when developing a material for a medical application. In this work, conventional sterilization methods (steam-, radiation- and gas sterilization) were investigated regarding the influence on the degree of swelling, mechanical performance and chemical effects on the poly 3-sulfopropyl acrylate potassium (pAESO_3_) hydrogel, which is a promising representative for biomedical engineering applications. In summary, no significant changes in the gel properties were observed after sterilization, showing the potential of the selected hydrogel for biomedical applications.

## Abstract 2

AESO_3_-PEGDA hydrogels are well suited for sterilization by common sterilization methods, enabling their use in biomedicine.

## Graphical abstract

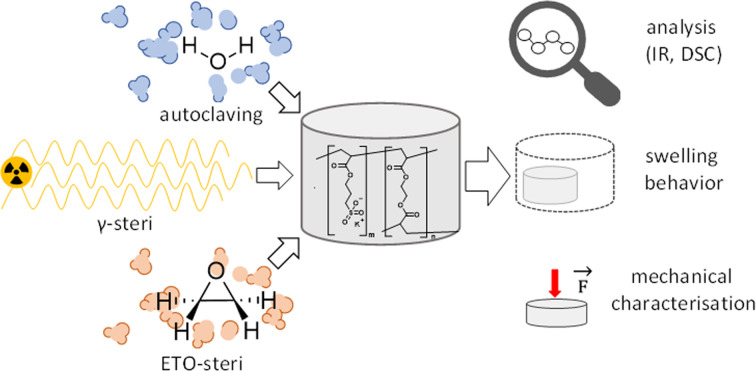

## Introduction

Alongside several other abilities, such as high biocompatibility, resistance to rheological stresses, antimicrobial activity and self-healing capabilities, the high swelling ability is one of the valued properties of hydrogels. This makes this class of materials remarkably versatile for biomedical applications. In particular, hydrogels based on polymerized ionic liquids have been shown to be promising for lubricating coatings or wound dressings, and as drug delivery systems, which was shown before for polymerized acrylate-based sulfonates.^1–3^ Although previous studies on biocompatibility and antibacterial properties suggest further applications in the biomedical field, sterilizability is a mandatory parameter for further clinical uses. Sterilization processes are much more aggressive than simple disinfection. In the literature, the sterilization of sensitive materials is described as one of the most difficult tasks to complete, due to the effects of high temperature or radiation on their structure. Possible consequences could be the hydrolysis and degradation of the polymer during the process.^4,5^ There are many examples in the literature that show how strongly the sterilization method can influence the properties of gels.^6–8^ This often leads to a reduction in water absorption capacity or changes in mechanical properties.^9,10^ As sterilization aims to completely destroy microorganisms and pathogens, the treatment is usually chemically aggressive and can therefore have a negative impact on the material to be sterilized.

In previous studies, poly-(3-sulfopropyl acrylate potassium) crosslinked with PEGDA (pAESO_3_) has shown good mechanical resilience with high compressibility paired with non-excessive swelling behaviour, good cell adhesion and biocompatibility, making it promising in the context of biomedical applications, *e.g.* as a drug delivery system for ion exchange controlled release.^2,3^ In this context, there is great interest in investigating how common sterilization protocols affect the properties of this particular material, as sterilisability is an important process in the use of medical materials. A few studies on hydrogels based on acrylates/bisacrylates have already been published in the literature, which have achieved similar results or have to struggle with some challenges, such as a decrease in water absorption capacity or changes in the elasticity mode.^8,11^ In order to assess the extent to which pAESO_3_ hydrogels are resistant to sterilization, three relevant sterilization methods in the field of biomedical device manufacturing, autoclaving, ethylene oxide (ETO) gassing and gamma irradiation, were tested on pAESO_3_. The influence on the swelling properties as well as on the mechanical conditions and the composition in general was investigated.

In particular autoclaving is frequently used in research laboratories and for medical instruments. It is a fast, inexpensive and widely available method. Another major advantage of autoclaving is that there are no toxic residues to be expected as in the case with *e.g.*, ETO sterilization. However, autoclaving is associated with the disadvantage that, in the case of hydrogels, the saturated water vapor atmosphere can lead to uncontrolled water uptake. In addition, steam and heat can drastically alter the mechanical and chemical properties of many biomaterials. Including synthetic ones, in particular when it comes to materials degradable or susceptible to hydrolysis.^12^ Overall, autoclaving has a high penetrability but takes long time to implement.^13,14^

ETO, as the second relevant sterilization method, places high demands on laboratory equipment in terms of safety and is time consuming. Moreover, ETO sterilization requires gas-permeable packaging. Therefore, the hydrogel samples must be dried prior to sterilization process in order to avoid changes in the material due to uncontrolled water loss over the transport period or sterilization process and to ensure comparability. Gamma irradiation is particularly practical as the devices and products to be sterilized can be stored in various containers, *e.g.*, glass bottles, as the radiation treatment penetrates a range of materials. Especially for hydrogels, this enables the sterilization of already hydrated or swollen samples in sealed bottles or beakers, avoiding the need for additional treatments or drying and rehydrating steps. However, the radiation energy, usually in a range of several kGy, can damage the polymer-based material. With gamma irradiation, sterilization without toxic residues can be assumed with a high penetrating power. Unfortunately this method often affects the physiochemical and structural properties of the material.^15^

Other sterilization techniques are also of high relevance in everyday medical practice *e.g.*, disinfection using EtOH, plasma-based sterilization and electron beam sterilization. However, we have not opted for these methods due to the fact that EtOH must diffuse completely into the hydrogel for complete sterilization. This leads to the need for complete removal when it comes to medical application. Plasma and ion beam-based sterilization mainly affect the surface of the hydrogel, whereas microbes are able to survive inside the gel bulk.

## Materials and methods

### Chemicals

Poly(ethyleneglycol)diacrylate (PEGDA, *M*_n_ = 575; SigmaAldrich), *N*,*N*,*N*′,*N*′-tetra-methylethylenediamine (TMEDA; ≥99.5%; VWR International), ammonium persulfate (APS; 98%; Acros Organics), 3-sulfopropylacrylate potassium salt (AESO_3_; 98%; SigmaAldrich) were used as received.

For 1 L of PBS 8.00 g NaCl, 0.20 g KCl, 1.44 g Na_2_HPO_4_·2H_2_O and 0.12 g KH_2_PO_4_ are dissolved accordingly in ultrapure water and the pH of 7.4 is adjusted with 0.1 M HCl.

### Hydrogel synthesis

The hydrogel was synthesized by radical polymerization as previously described.^2,3^ 3-Sulfopropylacrylate potassium (AESO_3_) and PEGDA (*M*_n_ = 575, 2 mol%) were dissolved in ultrapure water (AESO_3_ : PEGDA = 49 : 1). Subsequently, ammonium persulfate (APS) solution (0.1 mol%) was added and the reaction mixture was degassed for 15 min. After degassing, tetramethylethylene diamine (TMEDA, 1.9 mol% of the total monomer concentration, TMEDA : APS = 5 : 1) as catalyst was added and the mixture was filled into was filled into syringes (10 mL) and rested for 24 h at 22 ± 2 °C.

### Gamma sterilization

Gamma sterilization was performed by an external contractor (BBF Sterilizations Service GmbH, Kernen, Germany). Samples as obtained after synthesis without further treatment were placed in screw cap glass vials and exposed to a radiation dose ≥25 Gy, using a Co-60 radiation source.

### Ethylene oxide sterilization

ETO sterilization was performed by an external contractor (Medicoplast International GmbH, Illingen, Germany). Prior to sterilization, samples were dried at room temperature on air for 48 h and placed in a gas-permeable well plate, which was sealed in standard sterilization bags. According to the contractors information, samples were preconditioned for 16.75 h at 35–45 °C and 45–80% relative humidity. Following, samples were exposed to ETO gas at 44.5 °C and 191 mbar with 18 kg ETO mass. Finally, desorption took place for 3 days at 35–45 °C.

### Autoclaving sterilization

Autoclaving was carried out in a Tuttnauer 2540 ELV autoclave (Tuttnauer, Breda, Netherlands). Samples were placed in glass vials, which were loosely covered using aluminum foil. The samples were heated to 120 °C in 20 min in the autoclaving chamber, and the temperature was maintained for 20 min. After autoclaving, the samples were cooled to ambient conditions.

### Mechanical testing

Uniaxial compression testing was performed under ambient conditions using a ZwickiLine ZN 2.5 testing machine (Zwick/Roell, Ulm, Germany) equipped with a 1 kN load cell and two compression plates at a crosshead speed of *v* = 2 mm min^−1^ with *n* = 5. Force was measured as a function of compression. The compressive modulus (*E*) was determined in the linear elastic region between 0.05 to 0.25% compression by linear regression. From the collected data, the compression at break (*ε*_B_) and ultimate compression strength (*σ*_m_) were extracted. For the rehydration of the specimens for mechanical testing of autoclaving and ETO influence, specimens were rehydrated by placing the samples on a hydrated foam mat for several hours until adequately soft and weighed afterwards to determine the missing deionized water (DW) volume. Subsequently, the missing DW volume was added using a micropipette directly on the specimen. Cyclic mechanical testing was performed using the same set-up, applying up to 20% compression over *n* = 10 cycles. Parameters used were a preload of *σ* = 0.025 MPa, a crosshead speed for loading *v* = 5 mm min^−1^ and a crosshead speed for unloading of *v* = 10 mm min^−1^. Unloading was performed until *σ* = 0.025 MPa, after which the next cycle started.

### Gravimetric swelling experiments

Water uptake of pAESO_3_ hydrogel before and after sterilization was studied gravimetrically using PBS-buffer at 36 ± 1 °C as a function of time in a strainer. The weights of the swollen gels were determined at different intervals, after dripping through the strainer and dabbing the samples off, until the equilibrium swelling was attained. The degree of swelling (*q*_*t*_) was calculated according to the following equation:1
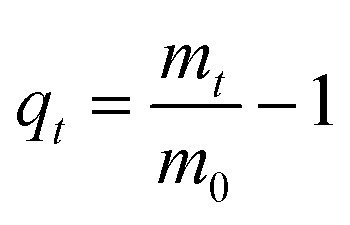
With *m*_0_ being the initial weight and *m*_*t*_ being the final weight of the gel at timepoint *t*. Swelling experiment has been performed in triplicates and values are given as mean ± standard deviation. The equilibrium water content (EWC) was calculated with the following term:2
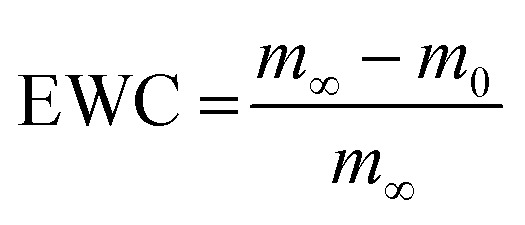
with *m*_∞_ being the mass of the swollen gels in equilibrium state and *m*_0_ being the initial dry mass at the time *t* = 0.

The kinetics of the polymer swelling was investigated in more detail an according to the calculations of one of our previous studies.^16^ In short, the process can be described as a second order process:3
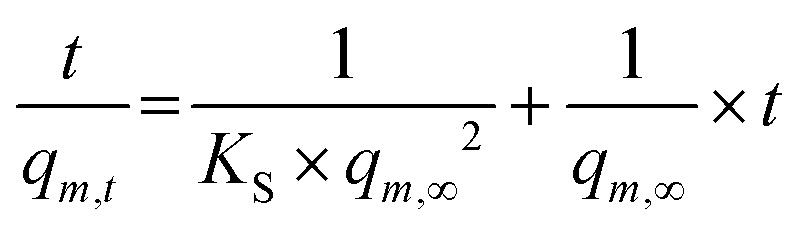


From the obtained slope and the y-intercepts of the linear regression of the experimental data, theoretical values of the initial swelling rates and the swelling rate constants *K*_S_ were calculated.

### Fourier transform infrared spectroscopy-attenuated total reflectance

Data were collected in the range of *ν̃* = 400 cm^−1^ to 4000 cm^−1^ with a resolution of *ν̃* = 4 cm^−1^ averaged over 32 scans in reflection mode using a Bruker Alpha T Nicolet 380 FT-IR with a Smart Orbit ATR-Unit. Atmospheric compensation has been performed. All spectra were subsequently baseline corrected and finally normalized with OPUS vector-normalization-function for better visualization.

### DSC experiments

Thermal analysis was carried out using a DSC 1 Star^e^ system (Mettler Toledo, Greifensee, Switzerland). Conventional calibration was performed using highly pure indium and zinc. Samples were heated from *T* = −10 °C to 310 °C with a heating rate *q* = 10 K min^−1^. Samples were dried prior to DSC analysis and sample weights were in the range of 3–10 mg.

### Statistical analysis

All data are given as mean ± standard deviation. For statistical analysis of swelling rate constant *K*_S_, initial swelling rate and mechanical parameters *E*, *ε*_B_ and *σ*_B_, two tailed *t*-tests of the means *versus* unsterilised reference have been performed. For statistical analysis, GraphPad Prism software (V5.04, GraphPad Software Inc., Boston, MA, USA) has been used. Significances are given at a significance level of *P* < 0.05 and marked with an asterisk.

## Results and discussion

### Synthesis of the hydrogels

**Fig. 1:**
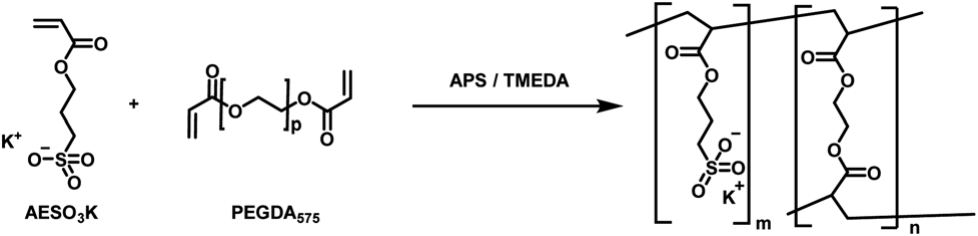
Synthesis of AESO_3_-PEGDA_575_-hydrogels with APS and TMEDA initiator system for radical polymerization.

Within this work, a hydrogel containing sulfonate groups was used. This system has shown good mechanical resilience with high compressibility paired with non-excessive swelling behaviour and good cell adhesion and biocompatibility properties.^2^ Furthermore, the sulfonate function enables ion exchange controlled drug release properties, which are of high interest in the field of the development of innovative drug delivery systems and medical device coatings. In brief, the hydrogel used in this study was synthesized by free radical polymerization starting from the monomer AESO_3_, using PEGDA (*M*_n_ = 575) as crosslinker and APS/TMEDA as initiator system (Fig. 1). PEGDA was chosen as crosslinker as it allows for *in vivo* degradation, which is highly beneficial as the implanted system vanishes from the body, enabling reduced long-term side effects from the foreign body reaction.^17^

### Handling of the hydrogels according to sterilization method

In contrast to gamma sterilization, where samples may be stored and treated in sealed vessels, the samples for ETO sterilization and autoclaving were dried prior to sterilization and rehydrated after sterilization before further testing. This is due to the fact that ETO sterilization requires gas permeable packaging to allow for the penetration of ETO gas during the sterilization, but leads to uncontrolled water evaporation during the whole process chain. For autoclaving, the process is carried out under a saturated steam atmosphere, which may lead to uncontrolled water uptake. To avoid uncontrolled swelling of the samples, the hydrogel specimens ns were dried before and after the sterilization process. For the mechanical testing, to restore the initial swelling state, the samples were rehydrated by adding the weight difference of deionized water to restore the initial swelling state. Notably, direct exposure to water, *e.g.*, by adding the volume difference using a pipette, caused the samples to burst. We observed very quick swelling directly at the point of contact with the water, causing internal stress in the sample, leading to cracks. To avoid this, the samples were first slowly rehydrated by using a hydro foam mat and then, if necessary, additional water was added to achieve the desired degree of swelling.

### Degree of swelling characterization

**Fig. 2:**
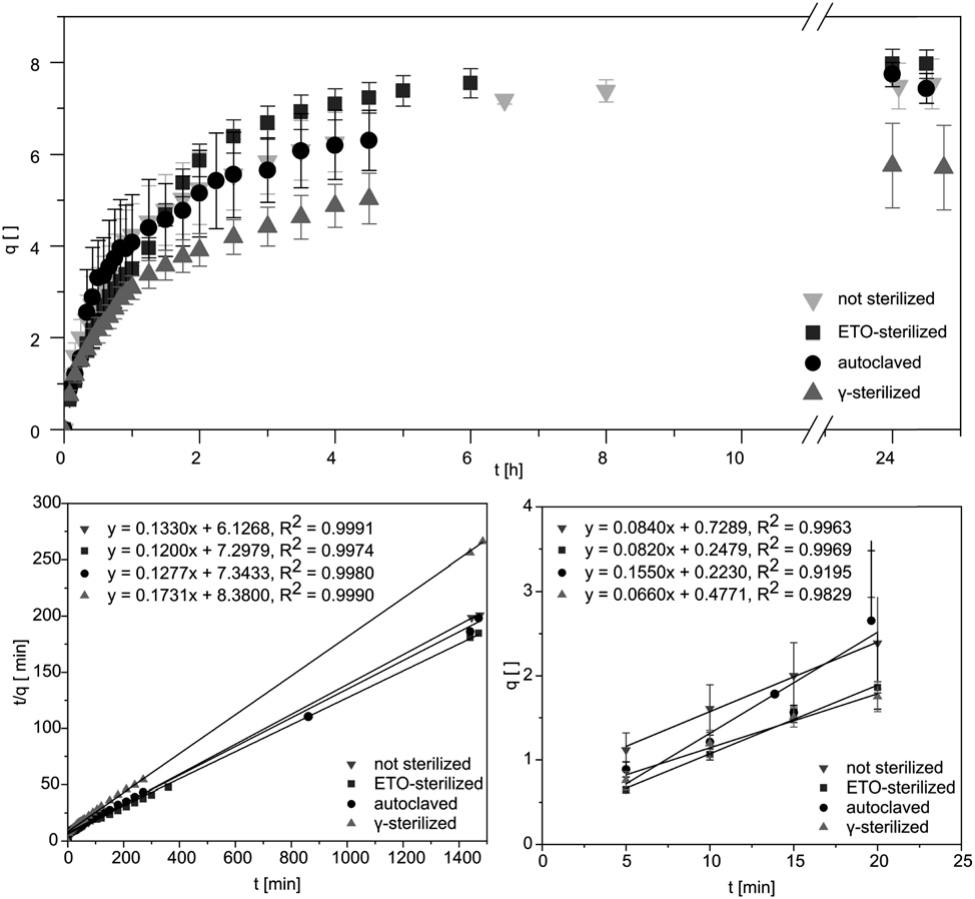
Degree of swelling *q*_*t*_ of AESO_3_-PEGDA_575_-hydrogels without and after sterilization. Diagrams used for further calculations exemplary for the swelling experiment. Linear fits to obtain the initial swelling rate and the swelling rate constant *K*_S_.

Studying the swelling behaviour is a feasible way to quickly detected material defects and serves to provide a fast characterization of the material. Data for equilibrium swelling of the synthesized hydrogels at 36 ± 1 °C are shown in Fig. 2.

Investigation of swelling behaviour was performed in phosphate buffered saline (PBS). Untreated pAESO_3_ gel showed a degree of swelling up to *q* = 7.5 ± 0.5 within 390 min. Similar results were received for autoclaved hydrogels (Table 1). After ETO sterilization the samples showed a slightly faster swelling, but achieved a similar degree of swelling in equilibrium. From our data it became obvious that only gamma sterilization had the effect that the treated hydrogels showed reduced swelling up to *q* = 5.7 ± 0.9 within 270 min. It has been shown in the literature that the mechanical properties and swelling of hydrogels can be controlled by the use of different types of crosslinking molecules and by controlling the crosslinking density.^18^ It can therefore be assumed that the rather aggressive sterilization with high-energy gamma radiation leads to further linkages between monomer and crosslinker and thus increases the crosslinker density. As a result, less water can be absorbed and the degree of swelling is decreased. However, as the samples do not have to be dried for gamma sterilization, this method allows a more feasible handling of the hydrogels. Similar results were obtained by Kanjickal *et al.* when they sterilized their PEG hydrogels with ETO gassing, gamma radiation and H_2_O_2_. Sterilization with the first two methods mentioned led to a loss of swelling of the samples. They also suggest that this is due to the formation of free radicals, which can lead to further cross-linking in the polymer network. While sterilization with H_2_O_2_ led to the breaking up of the crosslinks and a higher degree of swelling.^7^ Eljarrat-Bienstock *et al.* even observed this effect after the autoclaving of their hydrogel sponges based on hydroxyethyl methacrylate (HEMA) crosslinked with ethylene glycol dimethylacrylate (EGDMA) samples. Here, the samples demonstrated a 10–12% decrease in their water absorption.^11^

**Table 1:** Swelling parameters as extracted from swelling experiments (Fig. 2). Values for initial swelling rate and *K*_S_ for sterilized samples versus unsterilized reference were n.s. (*P* < 0.05). Significance was determined using two-tailed t-tests

Sample	*q*_*m*,∞_	Initial swelling rate [min^−1^]	*K*_S_ [10^−1^]	EWC
Not sterilized	7.5 ± 0.5	0.084 ± 0.02	1.33 ± 0.06	0.879 ± 0.007
Autoclaved	7.4 ± 0.3	0.115 ± 0.037	1.28 ± 0.05	0.881 ± 0.004
ETO	7.9 ± 0.3	0.082 ± 0.002	1.20 ± 0.05	0.888 ± 0.005
Gamma	5.7 ± 0.9	0.066 ± 0.004	1.73 ± 0.26	0.850 ± 0.023

From the obtained slope and the *y*-intercepts of the linear regression of the experimental data, theoretical values of the initial swelling rates and the swelling rate constants *K*_S_ could be calculated (Fig. 2). The swelling rate describes the absorption of water of the hydrogel and the swelling process. It depends on the surface area to volume ratio and is also affected by the initial swelling status of the hydrogel as well as the structure of the gel. Based on thermodynamic theories the focus is more on initial states and equilibrium end-states.^19^ However, focused control of hydrogel swelling rates based on hydrogel structure has potential for application in both drug delivery and biomechanical actuation.^19^ The initial swelling rate describes the initially linear increase in the swelling curve within the first 20 minutes. By means of these parameters, conclusions can be drawn to evaluate the effects of the sterilization processes on the material at a molecular level, as cross-linking or degradation would directly influence the swelling behaviour of the hydrogel.

Additionally, the swelling rate constant was identified and the equilibrium water content (EWC) was calculated using eqn (2), representing water that was absorbed from the hydrogels in the equilibrium state, as summarized in Table 1. The EWC represents water that was absorbed from the hydrogels in the equilibrium state. It is calculated by the ratio of the mass of the gel in equilibrium subtracted by the dry mass to the mass of the gel in equilibrium.

### Mechanical characterization

**Fig. 3:**
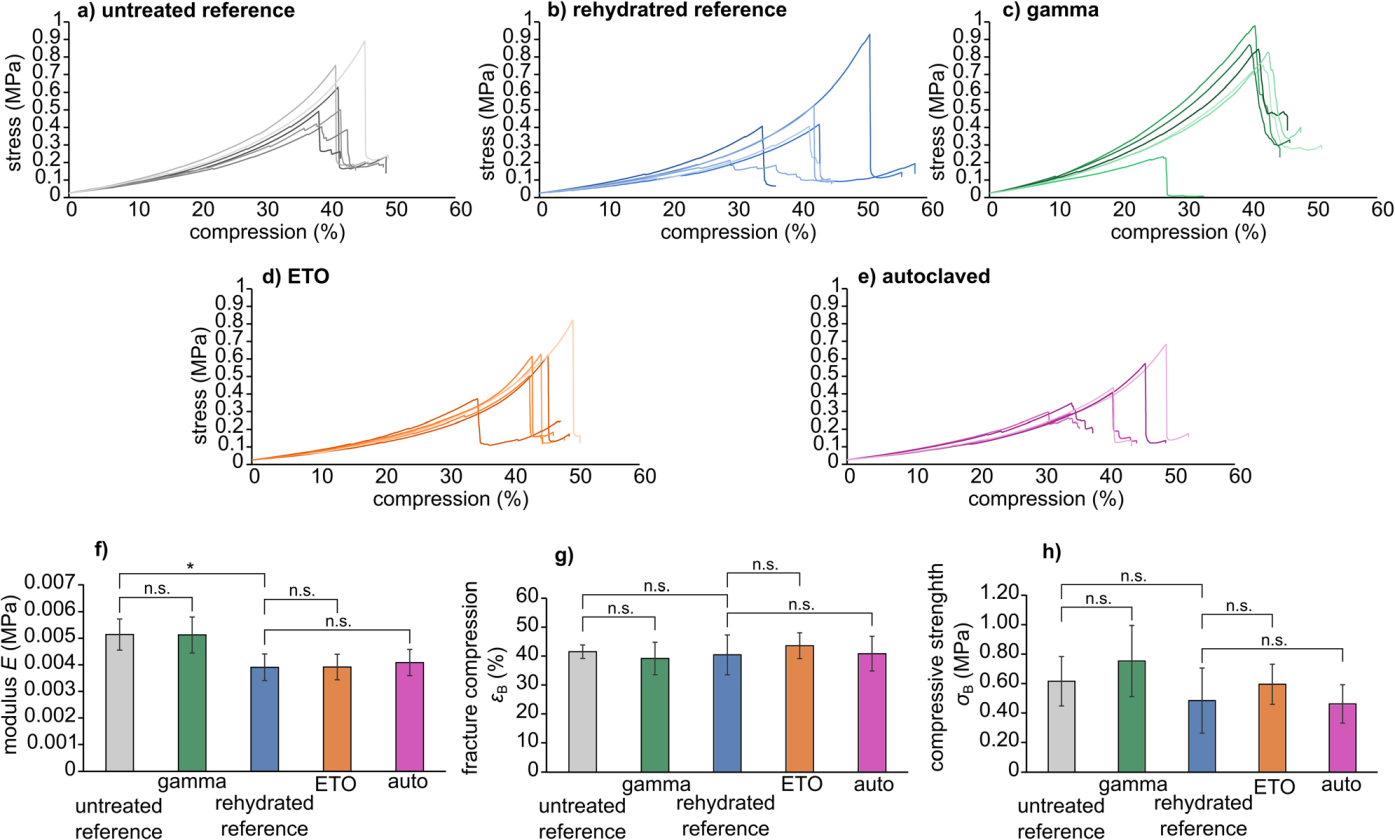
Compression curves of AESO_3_-PEGDA_575_-hydrogels without and after sterilization as function of stress [MPa] *versus* compression [%] (a–e) and bar graphs of average values of modulus *E* (f), fracture compression *ε*_B_ (g) and compressive strength *σ*_B_ (h). Asterisks indicate significance (*P* < 0.05), n.s. indicate no significance (*P* < 0.05) as determined by two-tailed *t* test.

The stress–compression curves of the sterilized samples are shown in Fig. 3. In particular, the modulus *E*, fracture compression *ε*_B_ and compressive strength *σ*_B_ are viable parameters for determining the influence of the sterilization method on the molecular integrity of the hydrogel, leading for example to chain breaks or crosslinking. For the presented specimens, statistical analysis showed that rehydration leads to a slight decrease in compression modulus *E* compared to the untreated reference (*E*_Ref_ = 0.0051 ± 0.0005 MPa *versus E*_rehydrated_ = 0.00039 ± 0.0005 MPa with *P* = 0.0078). However, no significant differences were found between the respective reference groups and the sterilized samples (untreated reference *versus* gamma sterilized samples, and rehydrated reference *versus* ETO sterilized samples and samples sterilized by autoclaving). Thus, we conclude that the three sterilization methods do not have a major effect on the material's mechanical properties. The mechanical parameters extracted from compression curves are given in Table 2.

**Table 2:** Mechanical parameters as extracted from stress–compression curves

	Reference	Gamma	Rehydrated	ETO	Autoclave
*E* (MPa)	0.0051 ± 0.0005	0.0051 ± 0.0007	0.0039 ± 0.0005	0.0039 ± 0.0005	0.0041 ± 0.0005
*ε*_B_ (%)	41.5 ± 2.3	39.2 ± 5.6	40.4 ± 6.9	43.6 ± 4.5	40.8 ± 6.0
*σ*_B_ (MPa)	0.61 ± 0.17	0.75 ± 0.24	0.48 ± 0.22	0.59 ± 0.14	0.46 ± 0.13

**Fig. 4:**
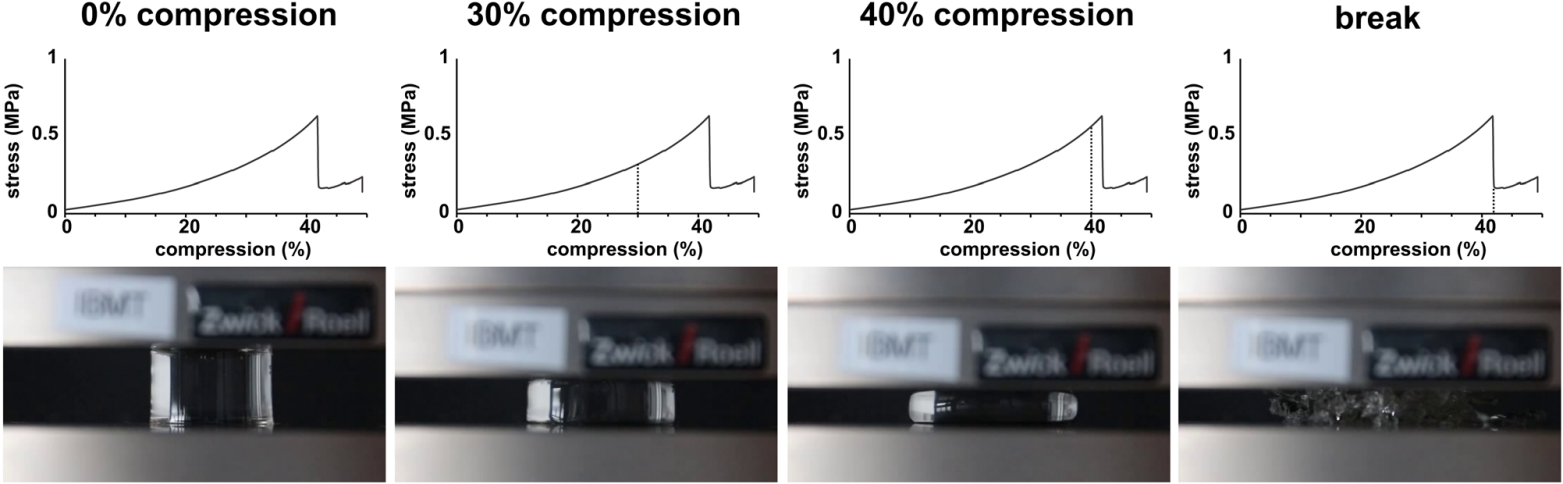
Exemplary images of the compression behaviour of pAESO_3_-PEGDA_575_ hydrogel reference at 0%, 30%, 40% compression and at compression at break (42%). The dashed lines show the respective compression (%) for the corresponding video capture frame.

All samples showed similar compression behaviour as shown exemplarily for the untreated reference in Fig. 4 for 0%, 30%, 40% compression and directly at *ε*_B_. Overall, the materials deform elastically until spontaneously fracture. Where the samples did not show partial breaking during compression in the video capture, some samples, however, showed the formation of cracks visible as steps in the stress–compression curves (Fig. 3). Still, even with these small cracks, the samples were mechanically highly resilient. As this was comparable for all tests, irrespective whether sterilized or not, only one set of images is provided.

Compared to other hydrogel materials reported in the literature, these results are of great significance. Gelatine methacryloyl hydrogels have already shown a significantly smaller compressive modulus after autoclaving.^8^ This behaviour was also observed after radiation treatment. Shi *et al.* also discovered a correlation between the mechanical changes and the dose of radiation applied to their polyvinyl alcohol-based gels. The compressive strength and the compressive modulus increase with increasing radiation dose, but a decrease is observed at doses higher than 100 kGy.^20^

**Fig. 5:**
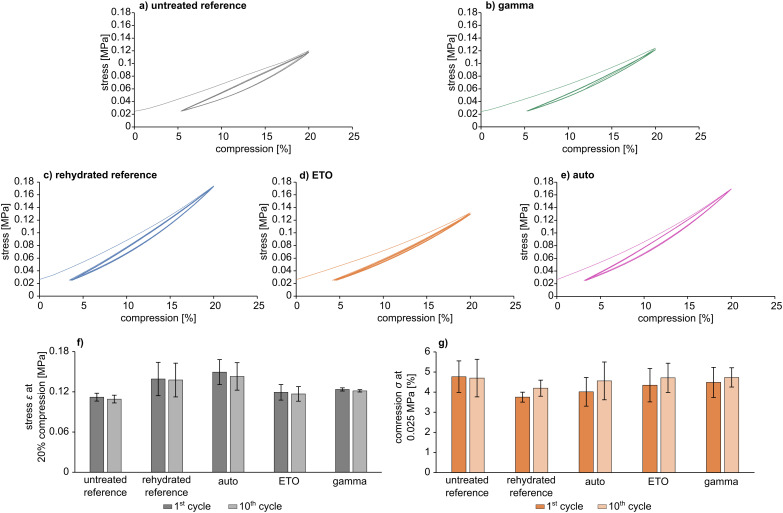
Exemplary cyclic compression curves for pAESO_3_ hydrogel (a and c) and pAESO_3_ hydrogel after sterilization (b, d and e) and characteristic parameters stress *ε* at 20% compression (f) and compression *σ* at 0.025 MPa (g) calculated as mean value for *n* = 3.

Cyclic tests (Fig. 5) showed comparable behaviour to the uniaxial mechanical test under load for all samples (Fig. 3). In particular, pAESO_3_ hydrogel did not show any creep behaviour after 10 cycles with 20% compression, even after sterilization. However, specimens that exhibited partial fracture, which was also observable in the uniaxial test and can be recognised as peaks in the stress–compression curves, were not included in the material evaluation, although the material still responded to the cyclic test. Differences in *ε* and *σ* were observed between the individual samples, which can presumably be attributed to intrinsic material differences. Nevertheless, the values of stress *ε* at 20% compression and compression *σ* at 0.025 MPa were in a comparable range for all cycles, and it was observed that sterilization did not affect the mechanical strength.

### Fourier transform infrared spectroscopy

**Fig. 6:**
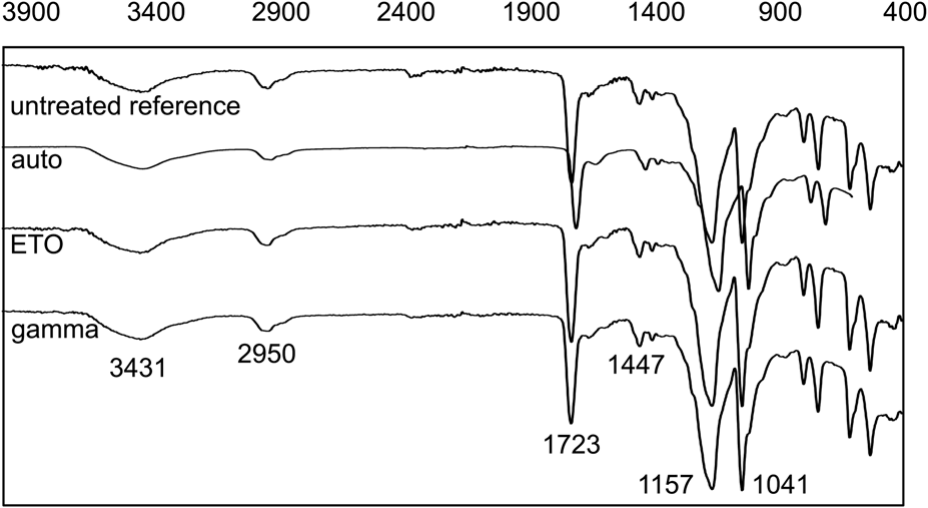
FT-IR-spectra of dried AESO_3_-PEGDA-hydrogels without and after sterilization. Wavenumbers (cm^−1^) for relevant bands are shown.

The results of the FT-IR measurements are shown in Fig. 6.

The characteristic bands with comparable intensity can be clearly seen. Differences in the FT-IR were not observed. Potential influences on the chemical structure of the hydrogels due to sterilization are, in particular, additional crosslinking of unreacted PEGDA or the polymer network itself through chain scission and recombination reactions. As no observable changes in the IR spectra, such as additional signals or altered signal intensities, were detected, we conclude based on FT-IR analysis that the selected sterilization methods have no pronounced influence on the chemical composition of the material.

The previously assumed fundamental changes in the gel structure due to gamma irradiation cannot be confirmed by the IR measurements either. It can therefore be assumed on the basis of this additional analysis method that the type of sterilization has no influence on the basic composition of the gel samples. An overview of the assignment of the signals to the respective functional groups can be found in Table 3.

**Table 3:** Assignment of the IR bands to the respective chemical groups in the AESO_3_-PEGDA_575_ hydrogel^21^

Identification	Wavenumber (cm^−1^)	Chemical group
1	3431	O–H from the intermolecular and intramolecular hydrogen bonds
2	2950	C–H from alkyl groups
3	1723	C=O
4	1447	CH_2_
5	1157 & 1041	C–O–C
6	880–700	C–H (di- or trisubstituted)

### Thermal analysis

**Fig. 7:**
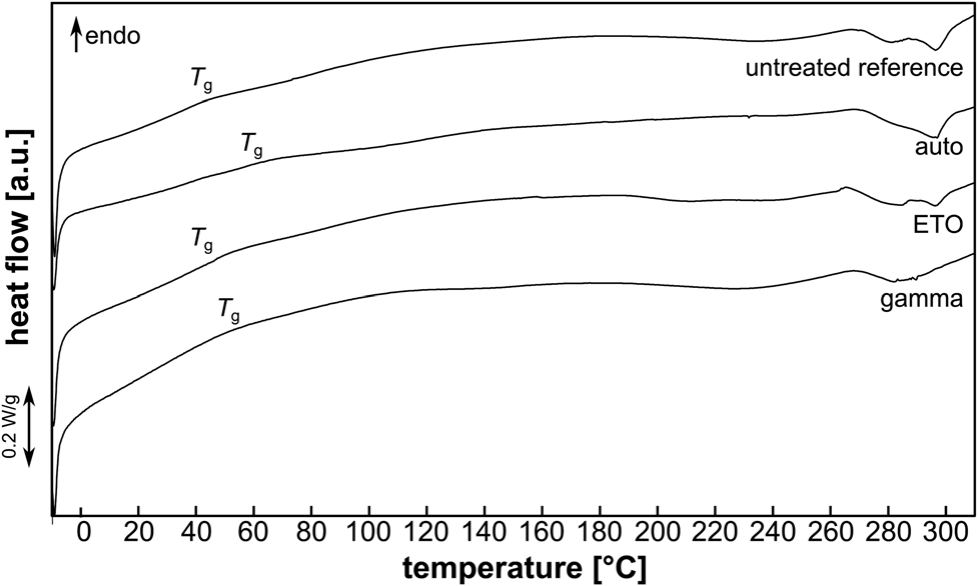
Exemplary heating curves of pAESO_3_-PEGDA_575_ hydrogel without and after sterilization. Position of glass transition (*T*_g_) is indicated.

DSC analysis of the pAESO_3_ hydrogel showed no characteristic melting or crystallisation processes that could be identified by peaks in the endothermic or exothermic range in the heating curve (Fig. 7).

However, when heated to approx. 270 °C, decomposition was observed, which became violent and rapid and vulcanisation of the hydrogel was observed when heated to 370 °C (not shown). The glass transition temperature *T*_g_ was comparable for the pAESO_3_ hydrogel before and after ETO sterilization (Table 4). Gamma sterilization and autoclaving led to a slight increase in *T*_g_.

**Table 4:** Glass transition (*T*_g_) values (1st heating cycle) for AESO_3_-PEGDA_575_ hydrogel without and after sterilization (*n* = 4)

	Reference	Auto	ETO	Gamma
*T*_g_ [°C]	47.5 ± 2.5	59.6 ± 2.6	45.9 ± 1.2	52.0 ± 3.2

This could be due to reduced chain mobility after hydrolysis (auto) or chain scission (gamma) of the PEDGA crosslinker, but this minor degradation did not show to affect mechanical or swelling behaviour (Fig. 2, 3 & 5).^22,23^

## Conclusions

Within this work, we showed that pAESO_3_ hydrogels are applicable for clinical relevant sterilization methods. Influence of sterilization on hydrogel properties was investigated with regard to mechanical characteristics and swelling behaviour. Our finding was that for ETO sterilization and autoclaving no significant influence on the hydrogel properties were found. However, a lower degree of swelling was detected for gamma-sterilization. The lower water uptake compared to the other sterilized samples may be caused from additional crosslinking of the polymer network by the energy rich radiation. To what extent the hydrogel polymer was affected, however, was below the detection limit of IR spectroscopy. As far as the mechanical properties are concerned, no changes in compressibility were detectable in the gamma-sterilized samples either. Moreover, sterilization did not show to affect the materials mechanical properties concerning cyclic mechanical load. Thermal analysis revealed a slight degradation caused by autoclaving, but this did not appear to have a strong negative impact on the general material properties, particularly the mechanical resilience. In summary, pAESO_3_ hydrogels are well suited for being sterilised by common sterilization methods.

## Data availability

Data for this article are available at Zenodo at DOI: https://doi.org/10.5281/zenodo.13384842.

## Author contributions

Conceptualization: J. R. & S. O.; data curation: J. R.; formal analysis: J. R. & S. O.; funding acquisition: N. G. & U. K; investigation: J. R. & S. O.; methodology: J. R. & S. O.; project administration: T. E.; resources: N. G. & U. K.; supervision: N. G. & U. K.; validation: all; visualization: S. O.; writing—original draft preparation: J. R. & S. O.; writing—review and editing: all. All authors have read and agreed to the published version of the manuscript.

## Conflicts of interest

The authors declare no conflict of interest.
